# Registered Clinical Trials Comprising Pregnant Women in China: A Cross-Sectional Study

**DOI:** 10.3389/fphar.2022.850080

**Published:** 2022-04-05

**Authors:** Yi Zhao, Guiping Du, Xiaofei Luan, Hui Yang, Qiongguang Zhang, Zhengfu Zhang, Subiao Wang

**Affiliations:** ^1^ Women’s Hospital, School of Medicine, Zhejiang University, Hangzhou, China; ^2^ Device Evaluation Center Zhejiang Medical Products Administration, Hangzhou, China; ^3^ Center for Food and Drug Inspection of NMPA, Beijing, China; ^4^ Zhejiang Medical Products Administration, Hangzhou, China

**Keywords:** clinical trials, pregnant women, drug development, drug safety, registration and post-marketing studies

## Abstract

**Background:** In this study, an investigation was conducted on clinical drug trials comprising pregnant women in China that provided data on the quantity, properties, source of funding, and geographical distribution regarding registration and post-marketing studies.

**Methods:** We conducted a cross-sectional descriptive study of clinical trials of pregnant women in China on 30 December 2021, and it was registered on the official Drug Clinical Trial Information Management Platform (ChiCTR) (http://www.chinadrugtrials.org.cn) established by the State Food and Drug Administration of China (Chinese FDA).

**Results:** This study encompassed 72 registered trials (0.46%, 72/15,539) for data analysis. Of these trials, 43.1% of trials were started between 2013 and 2016, and nearly half of the trials (48.6%) were completed. Industries were listed as the primary sponsor for 95.8% trials. Economically developed eastern China and northern China, accounting for 69.5% of the 72 registered trials, were the most frequently identified study locations. Regarding study designs of these trials, more than half of the trials (70.8%) were randomized, 61.1% were a parallel assignment, 33.3% were phase 3, and half of the trials (54.2%) were open label. In total, 23 trials met the requirements after excluding trials of cancer and/or of postmenopausal women, accounting for 0.15% of the 15,539 registered trials in the ChiCTR websites. Of the 72 clinical trials, 54 drugs for 18 indications were included. Of these indications, the highest proportion of the trials is osteoporosis (27.8%), followed by cancer (22.2%), assisted reproduction (13.9%), and other indications (13.9%).

**Conclusion:** This survey revealed a significant shortage of the development, evaluation, and safety trials of pregnancy-related drugs in China. Modifying or adding legislation and providing financial incentives may therefore encourage pharmaceutical companies to conduct additional clinical trials on pregnant women.

## Introduction

Although women during pregnancy demonstrate concerns with regard to drug safety, numerous women still use prescription drugs throughout pregnancy due to disease or abnormal reactions that may occur in pregnancy (e.g., hypertensive disorders of pregnancy or gestational diabetes) (Anoshchenko et al., 2020). Approximately 40–80% of women receive at least one medication during pregnancy (Meyer et al., 2021). However, the vast majority of newly marketed medications have not been evaluated with respect to pregnant women (Ye et al., 2019; Carnovale et al., 2021). Although pregnant women are often excluded from clinical trials due to safety and ethical concerns (Saito et al., 2017; Kreuder et al., 2020; Van Der Straten et al., 2020), clinical trials constitute the most effective way to evaluate preventive and therapeutic strategies (Ranawaka et al., 2018), and they were widely regarded as comprising the most crucial evidential source of efficacy and safety (Ruff et al., 2014). However, the obstetric studies registered at http://clinicaltrials.gov from 2007 to 2012 accounted for less than 10% of the total number of registries (Stockmann et al., 2014). A systematic evaluation in 2016 reported that in all validly registered drug clinical trials, only 0.32% were for drugs during pregnancy and only 4.4% of these clinical trials of pregnancy drugs included preplanned pharmacokinetic (PK) studies (Scaffidi et al., 2017).

In September 2013, the State Food and Drug Administration of China (Chinese FDA) promulgated the Announcement of the Drug Clinical Trial Information Management Platform (https://www.nmpa.gov.cn/xxgk/ggtg/qtggtg/20130906120001263.html); this required the acquisition of a clinical trial approval from the Chinese FDA and to register and provide information disclosure in http://www.chinadrugtrials.org.cn/index.html to conduct a clinical trial.

The objective of the present study, then, was to investigate the current status and specific characteristics of clinical trials encompassing pregnant women in China and to provide valuable insights into the drafting relevance of countermeasure policies for the government and for the evaluation of drug clinical trials that include pregnant women.

## Materials and Methods

### Reporting Guideline

This study was a cross-sectional report in which we followed the Strengthening the Reporting of Observational Studies in Epidemiology (STROBE) guidelines (Zeng et al., 2015).

### Search Strategy and Selection Criteria

Clinical trial data registered on the ChiCTR (http://www.chinadrugtrials.org.cn) websites were collected, and we employed the registry search function to search any of the following terms: “Obstetrics,” “Pregnancy,” “Pregnant,” “Fetus,” “Birth,” “Perinatal,” “Newborn,” “Postpartum,” “Prenatal,” “Maternal,” “Maternity,” “Mother,” or “Birth Outcome” between 1 September 2013 and 30 December 2021. This investigation also included clinical trials of traditional Chinese medicines, supplements, and vitamins.

### Data Extraction

Information publicly accessible on the ChiCTR websites included sponsors and registered projects, such as funding organizations, types, and locations; basic information of clinical trials, such as titles, study sites, and date of first registration; and study designs, such as indications, name and category of drugs, study phase, design type, sample size, and primary and secondary endpoints. Two independent investigators searched ChiCTR websites using the same search terms and reviewed all of the retrieved studies individually and independently. If their opinions did not agree, a third reviewer was consulted, and the registered projects were re-reviewed for consensus.

We implemented a joint-phase nomenclature for phases 1/2 and phases 2/3 and classified them as phases 1 and 2, respectively, because of their small numbers (one project in phases 1/2, two projects in phases 2/3). The data from all of the aforementioned trial-related information were extracted and compiled into an Excel worksheet for subsequent checks and analysis.

Our exclusion criteria were 1) study participants ≤12 years of age; 2) contraceptive drug; 3) males; 4) where the pregnant women constituted an exclusion criterion in clinical experiments. In March 2016, the general office of the State Council of China promulgated the “Opinions on implementing the Consistency Evaluation for the Quality and Efficacy of Generic Drugs” (http://www.gov.cn/zhengce/content/2016-03/05/content_5049364.htm) and required clinicians to register clinical drug trials on ChiCTR (http://www.chinadrugtrials.org.cn) websites. The marketing authorization of a generic drug is based on the proof of BE trials (Viprey et al., 2020), which requires manufacturers to certify that their generic pharmaceuticals are bioequivalent to brand drugs (Zhong et al., 2018), and BE trials are often executed with healthy volunteers and avoided with respect to women during pregnancy (Government of Canada, 2018). Therefore, we removed the BE trials from our analyses to discern the impact of BE on our study. The inclusion and exclusion criteria in our clinical trial included the exclusion of pregnant women as an excluded object of this study, and the research subjects for the indications were not strictly screened, including postmenopausal women and cancer patients.

### Data Analysis

Descriptive analyses were used, and primary sponsors were classified as the university, hospital, industry, or other sponsors. If different sites were analyzed in the same region, we were entered into the cumulative calculation for that region. Categorical data are reported as frequencies and percentages, and continuous variables as median and interquartile ranges. We assessed the differences between counts of categorical variables using the chi-squared test. Ordinary chi-squared analysis was applied for inspection when n ≥ 40 and T ≥ 5, whereas a calibrated chi-squared test was employed for inspection when n ≥ 40 and 1 ≤ T < 5. All of the analyses were executed using SPSS 20.0 software. *p* values < 0.05 were considered statistically significant.

## Results

### Screening and Included Trials

**FIGURE 1:**
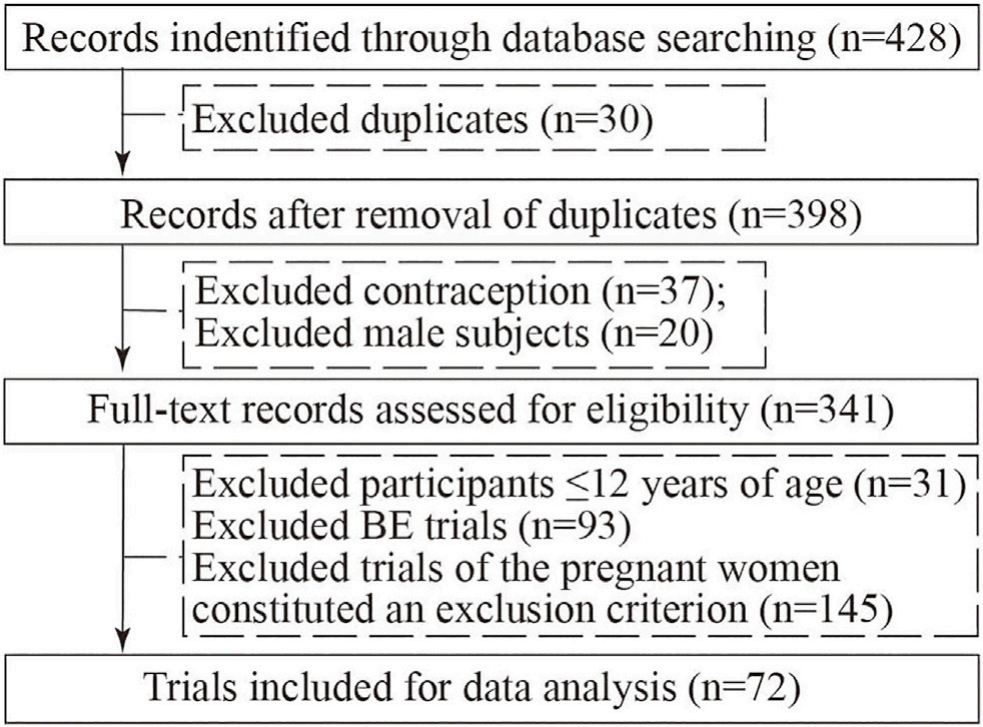
Flowchart of selection trials.

In our initial search, we found 428 clinical trials registered on ChiCTR websites on 30 December 2021, and after excluding duplicated trials, 398 trials remained; after carefully reviewing all the information, 37 contraception trials, 20 trials with male participants, 31 participants ≤12 years of age trials, 93 BE trials, and 145 trials where the pregnant women constituted an exclusion criterion in clinical experiments were excluded. Consequently, a total of 72 registered trials were ultimately evaluated (Figure 1).

### General Characteristics of Included Trials

The characteristics of the included trials are shown in Table 1. Thirty-one trials (43.1%) were started between 2013 and 2016, eighteen (25.0%) between 2017 and 2019, and twenty-three trials (31.9%) since January 2020. Nearly half of the trials (48.6%) were completed, followed by those actively recruited (33.3%), and those active, but not recruited (16.7%). The proportion of industry trials was 95.8%, and the majority was accounted for by the lead sponsor. Economically developed eastern China (51.4%) and northern China (18.1%) were the most frequently identified study locations. Drug classes were chemical (48.6%), biologics (40.3%), and natural (11.1%). Samples sizes were relatively large, with 62.5% of trials enrolling 100 or more participants. The median number of participants per trial was 216 (70–369).

**TABLE 1:** Characteristics of all included trials.

Variable	Subgroup	N (%)
Year	2013–2016	31 (43.1%)
	2017–2019	18 (25.0%)
	2020–2021	23 (31.9%)
Status	Active, not recruiting	12 (16.7%)
	Completed	35 (48.6%)
	Recruiting	24 (33.3%)
	Suspended	1 (1.4%)
Lead sponsor	University	2 (2.8%)
	Industry	69 (95.8%)
	Other	1 (1.4%)
Locations	Northern China	13 (18.1%)
	Eastern China	37 (51.4%)
	Northeast China	3 (4.2%)
	Central China	3 (4.2%)
	Southern China	5 (6.9%)
	Southwest China	9 (1.2%)
	Northwest China	2 (2.8%)
Drug classes	Chemical drugs	35 (48.6%)
	Biologic drugs	29 (40.3%)
	Natural medicine	8 (11.1%)
Enrollment	≤50	9 (12.5%)
	50–100	11 (15.2%)
	101–500	34 (47.2%)
	>500	11 (15.2%)
	Not applicable	7 (9.7%)

### Study Designs of Included Trials

More than half of the trials (70.8%) were randomized. The intervention models were parallel assignment (61.1%), crossover assignment (8.3%), and single-group assignment (30.6%). Regarding masking, over half of the trials (54.2%) were open label, followed by double-blind studies (44.4%) and single-blind (1.4%). Trial phases were as follows: phase 1 (16.7%), phase 2 (26.4%), phase 3 (33.3%), phase 4 (11.1%), and not applicable (12.5%). (Detailed data are depicted in Table 2.)

**TABLE 2:** Study design of all included trials.

Variable	Subgroup	N (%)
Allocation	Randomized	51 (70.8%)
	Non-randomized	21 (29.2%)
Intervention model	Crossover assignment	6 (8.3%)
	Parallel assignment	44 (61.1%)
	Single group assignment	22 (30.6%)
Masking	Single	1 (1.4%)
	Double	32 (44.4%)
	None (open label)	39 (54.2%)
Phases	Phase 1	12 (16.7%)
	Phase 2	19 (26.4%)
	Phase 3	24 (33.3%)
	Phase 4	8 (11.1%)
	Not applicable	9 (12.5%)

### Trial Characteristics and Study Design According to the Inclusion Criteria After Exclusion of Trials of Cancer and/or of Postmenopausal Women

We observed no significant differences between the two groups with respect to 1 year, drug classes, allocation, intervention model, masking, phases, and enrollment (*p* = 0.753, *p* = 0.698, *p* = 0.776, *p* = 1.000, *p* = 0.552, *p* = 0.936 and *p* = 0.661, respectively). Included pregnant women trials (41.3%) were principally initiated since January 2020, whereas excluded cancer and/or postmenopausal women trials (34.8%) primarily began during 2013–2016. (Detailed data are shown in Table 3.)

**TABLE 3:** Trial characteristics and study design according to the inclusion criteria after exclusion of trials of cancer and/or of postmenopausal women.

Variable	Subgroup	Included pregnant women (n = 72)	Excluding trials for cancer and/or of postmenopausal women (n = 23)	χ^2^/Fisher	*p* value
Year	-			0.633	0.753
	2013–2016	31 (43.1%)	8 (34.8%)	-	-
	2017–2019	18 (25.0%)	6 (26.1%)		
	2020–now	23 (31.9%)	9 (6.9%)		
Drug classes	-			0.819	0.698
	Chemical drugs	35 (48.6%)	13 (56.5%)	-	-
	Biologic drugs	29 (40.3%)	7 (30.4%)		
	Natural medicine	8 (11.1%)	3 (13.0%)		
Allocation	-			0.081	0.776
	Randomized	51 (70.8%)	17 (73.9%)	-	-
	Non-randomized	21 (29.2%)	6 (26.1%)		
Intervention model	-			0.291	1.000
	Crossover assignment	6 (8.3%)	1 (4.3%)	-	-
	Parallel assignment	44 (61.1%)	15 (65.2%)		
	Single group assignment	22 (30.6%)	7 (30.4%)		
Masking	-			1.282	0.552
	Single	1 (1.4%)	1 (4.3%)	-	-
	Double	32 (44.4%)	9 (39.1%)		
	None (open label)	39 (54.2%)	13 (56.5%)		
Phases	-			0.966	0.936
	Phase 1	12 (16.7%)	4 (17.4%)	-	-
	Phase 2	19 (26.4%)	6 (26.1%)		
	Phase 3	24 (33.3%)	7 (30.4%)		
	Phase 4	8 (11.1%)	4 (17.4%)		
	Not applicable	9 (%12.5)	2 (8.7%)		
Enrollment	-			2.609	0.661
	≤50	9 (12.5%)	4 (17.4%)	-	-
	50–100	11 (15.2%)	4 (17.4%)		
	101–500	34 (47.2%)	11 (47.8%)		
	>500	11 (15.2%)	4 (17.4%)		
	Not applicable	7 (9.7%)	0		

The characteristics of pregnant women trials according to the inclusion criteria and excluding trials for cancer and/or of postmenopausal women are given in Table 4. In total, 23 trials ultimately met our requirements, accounting for 0.15% of 15,539 registered trials in the ChiCTR websites, and 31.9% included 72 clinical trials.

**TABLE 4:** Trials on pregnant women according to the inclusion criteria after exclusion of trials of cancer and/or of postmenopausal women.

Accession number	Year	Status	Number of patient recruitment	Allocation	Masking	Phases	Intervention model	Pharmaceutical name	Drug classes	Primary purpose	Indications
CTR20171088	2017	Completed	252	Randomized	Double	Phase 3	Parallel assignment	rFSH injection	Biologic drugs	Safety/efficiency	ART
CTR20202525	2020	Recruiting	286	Randomized	Double	Phase 3	Parallel assignment	rFSH injection	Biologic drugs	Safety/efficiency	ART
CTR20150104	2015	Recruiting	12	Randomized	None (open label)	Phase 1	Crossover assignment	Cefotetan disodium injection	Chemical drugs	PK/PD	Antimicrobial/anti-infection
CTR20192307	2020	Recruiting	39	Non-randomized	None (open label)	Phase 1	Single group assignment	Ceftazidime avibactam sodium injection	Chemical drugs	PK/PD	Antimicrobial/anti-infection
CTR20180961	2018	Active, not recruiting	96	Randomized	None (open label)	Phase 1	Parallel assignment	Gonadotropin injection	Chemical drugs	PK/PD	ART
CTR20190023	2019	Completed	60	Randomized	None (open label)	Phase 1	Parallel assignment	Triptorelin acetate injection	Chemical drugs	PK/PD	ART
CTR20201374	2020	Recruiting	374	Randomized	None (open label)	Phase 2	Parallel assignment	rFSH-CTP injection	Biologic drugs	Safety/efficiency	ART
CTR20171142	2017	Completed	1740	Randomized	Double	Phase 3	Parallel assignment	rFSH injection	Biologic drugs	Safety/efficiency	ART
CTR20132181	2014	Recruiting	180	Randomized	Double	Phase 2	Parallel assignment	Yangxue Runchang granule	Natural medicine	Safety/efficiency	Postpartum constipation
CTR20150202	2015	Completed	240	Randomized	Double	Phase 3	Parallel assignment	Dexmethylphenidate HCl extended-release capsules	Chemical drugs	Safety/efficiency	ADHD
CTR20160582	2016	Recruiting	2100	Non-randomized	None (open label)	Phase 4	Single group assignment	Xiaoer Huanglong granule	Natural medicine	Safety/efficiency	ADHD
CTR20202341	2020	Completed	70	Randomized	None (open label)	Not applicable	Single group assignment	Enema chloral hydrate	Chemical drugs	PK/PD	Sedative-hypnotic
CTR20212041	2021	Active, not recruiting	70	Non-randomized	None (open label)	Not applicable	Single group assignment	Oral chloral hydrate	Chemical drugs	PK/PD	Sedative-hypnotic
CTR20131897	2014	Completed	180	Randomized	Double	Phase 2	Parallel assignment	Ruxin tablets	Natural medicine	Safety/efficiency	Postpartum hypogalactia
CTR20200507	2020	Recruiting	50	Non-randomized	None (open label)	Phase 4	Single group assignment	Nusinersen sodium injection	Chemical drugs	Safety/efficiency	Spinal muscular atrophy
CTR20131398	2014	Completed	240	Randomized	Double	Phase 3	Parallel assignment	Ferrous (II)–glycine–sulfate complex capsules	Chemical drugs	Safety/efficiency	Iron deficiency
CTR20132538	2014	Completed	340	Randomized	Double	Phase 3	Parallel assignment	Dimemorfan phosphate granule	Chemical drugs	Safety/efficiency	Acute upper respiratory, tract infection, acute bronchitis, and pneumonia
CTR20140858	2015	Completed	900	Randomized	None (open label)	Phase 4	Parallel assignment	polyethylene glycol, rFSH Injection	Biologic drugs	Safety/efficiency	ART
CTR20201723	2020	Recruiting	1000	Non-randomized	None (open label)	Phase 4	Single group assignment	Desogestrel tablets	Chemical drugs	Safety/efficiency	Endometriosis
CTR20212511	2021	Active, not recruiting	120	Non-randomized	None (open label)	Phase 2	Single group assignment	SHR7280 tablets	Chemical drugs	Safety/efficiency	ART
CTR20181474	2018	Completed	30	Randomized	Double	Phase 2	Parallel assignment	LCZ696 tablets	Chemical drugs	Safety/efficiency	Heart failure in children
CTR20191979	2019	Recruiting	358	Randomized	Single	Phase 3	Parallel assignment	rFSH injection	Biologic drugs	Safety/efficiency	ART
CTR20212525	2021	Recruiting	108	Randomized	None (open label)	Phase 2	Parallel assignment	rFSH-CTP injection	Biologic drugs	Safety/efficiency	ART

Note: rFSH, recombinant human follicle-stimulating hormone; PK/PD, pharmacodynamic/pharmacokinetic; ART, assisted reproductive technologies; ADHD, attention-deficit hyperactivity disorder.

### Overview of Investigated Drugs

A summary of studied drugs is provided in Table 5. Of the 72 clinical trials, 54 drugs for 18 indications were included. Regarding indications, the highest proportion of the trials is for osteoporosis (27.8%), followed by cancer (22.2%), assisted reproduction (13.9%), and other indications (13.9%). Seven trials investigated the parathormone for the treatment of *osteoporosis*, and five trials investigated the follicle-stimulating hormone for assisted reproduction*.*

**TABLE 5:** Overview of investigated drugs.

Indication	Name and number of investigated drug
Analgesia/anesthesia/sedation	Chloral hydrate (2), phloroglucinol (1)
Hemophilia	Coagulation factor VIII (1), coagulation factor IX (1), fitusiran (1)
Cancer	Anastrozole (2), carrelizumab (1), anti-PD-1 antibody (1), Cervarix (1), famitinib malate (1), pembrolizumab (1), niraparib (1), Tiragolumab (1), RAD001 (1), palbociclib (1), MGD013 (1), IBI310 (1), lapatinib (1), FCN-437c (1), Cervarix (1)
Assisted reproduction	Follicle-stimulating hormone (5), Menopur (1), triptorelin acetate (1), orgalutran (1), GnRH antagonists (SHR7280) (1), FSH-CTP (1)
Anti-infective	Cefotetan (1), ceftazidime-avibactam (1), zidovudine (1)
Osteoporosis	Parathormone (7), RANKL (2), minodronic acid (4), denosumab (2), teriparatide (1), strontium ranelate (1), alendronate (1), blosozumab (1), odanacatib (1)
Climacteric syndrome	Kunyuning granule (2), Liuwei Dihuang Tanggan tablet (1), Fuchun granule (1), Danzhiqine tablet (1)
Endometriosis	Dienogest (1), medroxyprogesterone acetate (1)
Other	Prasterone (1), Yangxue Runchagn granule (1), methylphenidate hydrochloride (1), Xiaohuanglong granule (1), Ruxin tablet (1), nusinersen (1), ferrous (II)–glycine–sulfate complex (1), dimemorfan phosphate (1), recombinant human growth hormone (1), sacubitril/valsartan (1)

## Discussion

Clinical trials are critical to clinical practice and decision-making (Mastroianni and Kahn, 2001). However, pregnant women were excluded from the majority of drug trials (Mofenson et al., 2019), yet other very relevant and/or more recent references of “drug utilization” studies could be used to show that pregnant women often (need to) use medication (Lupattelli et al., 2014; Ceulemans et al., 2022). As several investigators have determined, most trials have been funded by large pharmaceutical concerns that had better financial and organizational resources and more experts in conducting trials (Laterre and François, 2015). However, for ethical reasons, bias, and regarding potential harm to the fetus, large companies often excluded pregnant women from clinical trials (Fisk and Atun, 2008; Saito et al., 2017; Scaffidi et al., 2017; Kreuder et al., 2020; Van Der Straten et al., 2020), and these results were also similar to the present study. Reliable data on the quantity, location, source of funding, and therapeutic area of the trials on pregnancy-related drugs were provided. However, this demonstrated that there are very few clinical trials in which pregnant women were selected as research subjects. This finding is consistent with that reported in other countries, where the authors considered pregnant women as “drug orphans” (Kass et al., 2000; Mccormack and Best, 2014; Ayad and Costantine, 2015). Even when the number of pregnant women in a clinical trial is sufficient, the number of trials on pregnant women registered in China remains deficient compared to other regions of the world (e.g., North Africa/the Middle East, Europe, and North America) where trials of pregnancy-related drugs are actively pursued and implemented (Scaffidi et al., 2017). All of the clinical trials in the present study were approved by the Chinese FDA, with 95.8% funded by industry, and only 0.46% (72/15,539) of the clinical trials included pregnant women. After removing trials of cancer and/or on postmenopausal women, only 23 trials (0.15%, 23/15,539) included pregnant women. This result also indicated that studies on pregnant women might require government intervention, subsidies, incentives, and additional technologic advancements (Mastroianni and Kahn, 2001).

Neither the investigators nor the patients were particularly enthusiastic about participating in clinical trials. Our study found that since the establishment of the National Medical Products Administration (NMPA) registration platform in 2013, the total number of drug clinical trials registered on the platform and the CHiCTR platform was 44,505, which was only 1/5 the number of valid clinical trials on international registration platforms over the year. The results of our study also showed that the number of trials for new drugs and the assessments of efficacy and safety of the drugs for the treatment of common pregnancy conditions were insufficient and that there were no PK studies conducted on pregnant women. This was potentially due to the uncertainty of both the public and healthcare providers with regard to teratogenicity and other potential negative impacts of investigational drugs on fetal development, rather than on the mother (Kass et al., 2000; Ahmed et al., 2018). Collectively, our data revealed that the introduction of new drugs to the population of pregnant women would be difficult and complex. The medications most typically given to pregnant women would still be prescribed off label, and we acknowledge that data regarding the appropriate dosage, efficacy, and safety for pregnant women are still insufficient.

Randomized controlled, masked, and appropriate patient population trials are critical components of high-quality clinical trials (Zwierzyna et al., 2018). In our study, the percentage of randomized trials (70.8%) was lower than in previous studies 84.1% (Chen et al., 2020); 90.7% (Chen et al., 2019), which may be due to the robust effect of clinical trials that entailed cancer treatment or prevention/genetic diseases. After removing such trials, 51 trials (91.1%, 51/56) were ultimately randomized.

### Strengths and Limitations

The essential advantage of this study was its surveillance of the clinical trial data of pregnant women registered on the clinical trial information platform in China using a systematic, unbiased approach. However, there were three significant limitations. First, since 6 September 2013, new clinical drug trials were required by the NMPA to be registered on the drug clinical trial registration and information disclosure platform. Therefore, previously registered trials may not have been included in our analysis. Second, although some pharmaceutical companies or clinical institutions have conducted clinical trials on pregnant women, they have not yet registered on this information platform. Third, we were also limited by the cross-sectional nature of our study, which restricted our analysis of the factors influencing results from pregnant women included in clinical trials.

## Conclusion

Due to concerns regarding the fetus, it is common for pregnant women to show reluctance toward their inclusion in clinical trials. However, pregnant women and their spouses collectively agree that medical treatment should be administered for illnesses during pregnancy and that clinical trials of drugs during pregnancy are important and need to be performed. This practice paradoxically increases the risk to fetuses of untested or subtherapeutic drug regimens in clinical practice (Zhao et al., 2021). Based on our findings, we posit that the prospect for developing and evaluating drugs in pregnant women may not be favorable unless appropriate policies and measures are put in place. For example, studies on pregnant women might require government intervention, subsidies, incentives, and technological advancements (Mastroianni and Kahn, 2001). However, bioethicists, pharmacologists, regulators, and researchers elucidated on the need to include pregnant women in clinical trials to improve knowledge regarding the safety, dosage, and long-term effects drugs on pregnant women in the past few decades (Lyerly et al., 2008; White, 2015; Payne, 2019). Medical associations and regulatory agencies in various countries have been advocating for the removal of obstacles for pregnant women to be included in drug clinical research (Lyerly et al., 2008; Allesee and Gallagher, 2011; Payne, 2019). The U.S. FDA passed the “Pregnancy and Lactation Labeling Final Rule” (PLLR, Final Rule) in 2014. The rule requires an evaluation of the available information about a product’s use in pregnancy, which is expected to advance the development and implementation of clinical research on pregnant women (Gruber, 2015). Meanwhile, the results from this study should not be viewed as disappointing as there are still studies carried out on pregnant women at certain intervals by sponsors and clinical institutions in China. Nevertheless, greater attention needs to be given to the use of marketed drugs on pregnant women and to prioritize studies on drug PK in pregnant women.

## Data Availability

The original contributions presented in the study are included in the article/Supplementary Material, further inquiries can be directed to the corresponding author.
